# Early Probiotic Supplementation of Healthy Term Infants with *Bifidobacterium longum* subsp. *infantis* M-63 Is Safe and Leads to the Development of *Bifidobacterium*-Predominant Gut Microbiota: A Double-Blind, Placebo-Controlled Trial

**DOI:** 10.3390/nu15061402

**Published:** 2023-03-14

**Authors:** Akari Hiraku, Setsuko Nakata, Mai Murata, Chendong Xu, Natsumi Mutoh, Satoshi Arai, Toshitaka Odamaki, Noriyuki Iwabuchi, Miyuki Tanaka, Takahisa Tsuno, Masahiko Nakamura

**Affiliations:** 1 Food Ingredients and Technology Institute, R & D Division, Morinaga Milk Industry Co., Ltd., 5-1-83, Higashihara, Zama 252-8583, Japan; 2 Department of Pediatrics, Matsumoto City Hospital, 4417-180, Hata, Matsumoto 390-1401, Japan; 3 Next Generation Science Institute, R & D Division, Morinaga Milk Industry Co., Ltd., 5-1-83, Higashihara, Zama 252-8583, Japan; 4 Department of neurosurgery, Matsumoto City Hospital, 4417-180, Hata, Matsumoto 390-1401, Japan

**Keywords:** term infant, gut microbiota, probiotics, *Bifidobacterium longum* subsp. *Infantis*, GI motility, short-chain fatty acids, secretory IgA

## Abstract

Bifidobacteria are important intestinal bacteria that provide a variety of health benefits in infants. We investigated the efficacy and safety of *Bifidobacterium longum* subsp. *infantis* (*B. infantis*) M-63 in healthy infants in a double-blind, randomized, placebo-controlled trial. Healthy term infants were given *B. infantis* M-63 (n = 56; 1 × 10^9^ CFU/day) or placebo (n = 54) from postnatal age ≤ 7 days to 3 months. Fecal samples were collected, and fecal microbiota, stool pH, short-chain fatty acids, and immune substances were analyzed. Supplementation with *B. infantis* M-63 significantly increased the relative abundance of *Bifidobacterium* compared with the placebo group, with a positive correlation with the frequency of breastfeeding. Supplementation with *B. infantis* M-63 led to decreased stool pH and increased levels of acetic acid and IgA in the stool at 1 month of age compared with the placebo group. There was a decreased frequency of defecation and watery stools in the probiotic group. No adverse events related to test foods were observed. These results indicate that early supplementation with *B. infantis* M-63 is well tolerated and contributes to the development of *Bifidobacterium*-predominant gut microbiota during a critical developmental phase in term infants.

## 1. Introduction

The establishment of a healthy gut microbiota in the early developmental stages of human life plays an important role in later health [1]. Bifidobacteria are important intestinal bacteria that provide a variety of health benefits for infants, including the production of vitamins and organic acids [2], maintenance of gut homeostasis [3], improved vaccination response [4,5], prevention from infection [6], suppression of gut inflammation [7], and allergy prevention [8]. Reduction of bifidobacteria in infants could cause a variety of disorders and has been associated with an increased prevalence of obesity, diabetes, metabolic disorders, and all-cause mortality in later life [9,10,11]. The formation of *Bifidobacterium*-dominant microbiota is delayed in infants born by cesarean section [12], in newborns who used antibiotics at birth [13], and in low-birth-weight infants. Low-birth-weight infants with delayed colonization of *Bifidobacterium* have been reported to be at high risk for sepsis and necrotizing enterocolitis [14].

Human milk oligosaccharides (HMOs), a complex mixture of over 200 molecular species including fucose and sialic acid, are the third most abundant solids in breast milk after lactose and lipids and are the selective growth factors of bifidobacteria. HMOs are not digested in the small intestine because they cannot be broken down by the infant itself; instead, they reach the large intestine, where they are selectively capitalized upon by intestinal bacteria such as bifidobacteria and *Bacteroides*. The *Bifidobacterium longum* subsp. *infantis* (*B. infantis*) microorganisms have metabolic pathways that allow them to preferentially uptake and utilize HMOs, such as fucosyl-lactose (FL) transporters, fucosidases, and sialidases [15], and they utilize HMOs for their own bifidobacterial growth. *B. infantis*, which becomes the predominant bacterial species in the gut due to HMOs in breast milk, benefits infant health by promoting the maturation of immune function, inhibiting excessive inflammation, enhancing gut barrier function, and increasing acetate production [16].

Administration of probiotics to infants could maintain mucosal barrier integrity, regulate appropriate bacterial colonization, and modulate immune function by promoting IgA secretion in the mucosa and suppressing intestinal inflammation [17]. Early postnatal use of probiotics such as *Bifidobacterium* spp. in low-birth-weight infants has been reported to be effective in preventing sepsis and necrotizing enterocolitis and establishing enteral nutrition early by the formation of a *Bifidobacterium*-dominant microbiota [18,19,20,21]. In clinical studies in which *B. infantis* was administered to preterm or undernourished infants, anti-inflammatory effects and growth-promoting effects on infant development were reported [10,22]. However, current guidelines from the European Society of Pediatric Gastroenterology, Hepatology and Nutrition and the American Academy of Pediatrics state that there is insufficient evidence to recommend the use of probiotics for all neonates [17].

*B. infantis* M-63 was isolated from the feces of infants [22] and has been shown to grow well in human breast milk due to its high capability to capitalize on HMOs [23,24] and its tolerance to lysozyme [25]. From clinical studies in which probiotics containing *B. infantis* M-63 and a mixture of several other species of *Bifidobacterium* and *Lactobacillus* were administered to infants and children, the following effects have been reported: early establishment of *Bifidobacterium*-predominant gut microbiota and suppression of intestinal staphylococcal colonization in low-birth-weight infants [19], suppression of dysbiosis and growth promotion in neonates with congenital gastrointestinal surgical conditions (CGISC) [26], improvement of symptoms of gastrointestinal discomfort such as overflowing milk and bloating in colicky infants [27], reduction in the duration of crying or agitation [28], prevention and alleviation of the onset of allergies such as seasonal rhinitis and atopic dermatitis [29], alleviation of functional constipation [3], and improvement of QOL in children with functional gastrointestinal disorders [30]. However, the effect of administering the M-63 strain alone to full-term infants during the early postnatal period has not been examined to date. In this study, the effects of *B. infantis* M-63 as a single probiotic on gut microbiota formation, intestinal environment, gastrointestinal function, and immune parameters in healthy full-term infants up to 3 months of age were investigated in a double-blind, randomized, placebo-controlled trial.

## 2. Materials and Methods

### 2.1. Study Population

Between October 2019 and August 2021, healthy women who were scheduled to give birth or deliver a healthy full-term baby at Matsumoto City Hospital were recruited and subsequently provided written informed consent for participation in the study. Eligible participants were apparently healthy children born within 7 days whose gestational age at birth was ≥37 weeks and ˂42 weeks. Exclusion criteria for mothers were as follows: mothers who were diagnosed with any severe liver, renal, cardiovascular, respiratory, endocrine, metabolic, or mental disease or planned to administer any other probiotic supplements to their infants during the study period. Mothers with gestational diabetes or gestational hypertension were not excluded. Exclusion criteria for infants were as follows: infants born with multiple births and infants born with medical complications such as small for gestational age (SGA), large for gestational age (LGA), blood, liver, heart, kidneys, digestive disease, or suspected immunodeficiency or who had exposure to any oral or intravenous antibiotics or who were judged to be inappropriate to participate in the trial by the principal investigator.

### 2.2. Study Design

This study was a single-center, placebo-controlled, double-blinded randomized trial of probiotics in healthy full-term infants conducted in Matsumoto City Hospital. This study protocol was performed in compliance with the Helsinki Declaration of 1975 as revised in 2013 and the Ethical Guidelines for Medical and Health Research Involving Human Subjects proposed by the Ministry of Education, Culture, Sports, Science and Technology and the Ministry of Health, Labor and Welfare; it was reviewed and approved by the Research Ethics Committee of Matsumoto City Hospital (27 September 2019). This study was registered on the UMIN Clinical Trials Registry (UMIN000038351).

Prior to the initiation of the study, the investigator recruited participants from mothers giving birth at Matsumoto City Hospital and explained the details of the study according to the informed consent form. After giving birth, mothers completed a written consent form as a surrogate, and the infants who met the eligibility criteria were stratified by randomization schemes based on delivery mode (vaginal delivery/cesarean section) or sex (male/female) and assigned to two groups. Stratified randomization was utilized because delivery mode and gender have been shown to be influencing factors on early intestinal gut microbiota [31]. The infants in each group were fed *B. infantis* M-63 (1 billion CFU/1.0 g of sachet) or placebo (sterilized dextrin only/1.0 g of sachet) daily from within 7 days after birth to 3 months after birth. The test foods were suspended in a small amount of sterile water in a sterilized feeding bottle at room temperature, and mothers fed the suspension to their infants using a feeding bottle or a sterilized medicine dropper. After giving birth, the mother received support at the hospital to promote breastfeeding as much as possible, and the infants who were judged to require supplementation with infant formula were begun on mixed nutrition. None of the infants used HMOs-fortified infant formula.

### 2.3. Infant Gastrointestinal (GI) Tolerability and Health Examination

Infant GI tolerability and health was assessed by mothers on a daily basis from Day 1 until 3 months of age during test food supplementation period. Mothers were instructed to record the following information about their infants in daily logs: consumption of the amount of test foods, intake of any oral antibiotics or medicines, any symptoms including episodes of fever (≥38 °C), and hospital visits. For the 7 days before 1 week, 1 month, and 3 months of age, mothers recorded in daily logs the number of breast milk-fed and infant formula-fed infants, number of stools, stool consistency, duration and episodes of crying, number of regurgitations and vomiting episodes after feeding. Consistency of stool was assayed by mothers using a modified Amsterdam infant scale [32] (4-point scale with 1: watery; 2: soft; 3: formed; 4: hard). Episodes of crying were defined as more than 30 min per day. Episodes of colic were defined as 3 h per day for at least 3 days per week [33]. The investigator examined infant health and physical growth (length, body weight, and head circumference) immediately after birth and during hospitalization after delivery, before starting the study, and at 1 month and 3 months of age and checked the daily logs recorded by mothers.

A safety evaluation was conducted on all participants who consumed the study food once or more. Throughout the study period, all adverse events related to subjective and objective symptoms were recorded in the daily logs. The degree of symptoms and the causal relationship were evaluated according to the revised “National Cancer Institute, Common Terminology Standards for Adverse Events Version 4.0, Japanese Translation JCOG Version”.

### 2.4. Fecal Sample Collection

Two types of fecal samples were prepared: fresh fecal samples were collected before intake and at 1 month of age, and fecal samples with the preservative solution, guanidine thiocyanate solution, were collected before intake, 1 week after intervention, and at 1 month and 3 months of age. Both fecal samples were collected from infants’ diapers in each stool collection tube (Techno Suruga Laboratory Co., Ltd., Shizuoka, Japan). Fresh fecal samples were collected within 24 h of the hospital visit and were stored in a foamed styrene box with cooling agent at home, whereas fecal samples with the preservative solution were stored at room temperature at home. Both samples taken at home were transferred to the hospital and stored in −20 °C freezers. After being transported to the laboratory with dry ice, they were stored at −80 °C before DNA extraction. Fresh fecal samples were used for the analysis of real-time PCR, pH, SCFAs, and biomarkers, whereas fecal samples with the preservative solution were used for 16S rRNA gene sequencing. All participant samples were blinded to the process and analysis.

### 2.5. Fecal DNA Extraction and 16S rRNA Gene Sequencing

Fecal DNA was extracted according to previously described methods [34]. In brief, 200 μL of fecal sample in GuSCN solution was lysed with glass beads (300 mg, 0.1 mm diameter) and 300 μL of lysis buffer (No. 10 buffer, Kurabo Industries Ltd., Osaka, Japan) using a FastPrep-245G homogenizer (MP Biomedicals LLC, Santa Ana, CA, USA) at a 5-power level for 45 s with 5 min cooling intervals on ice. After centrifugation at 12,000 rpm for 5 min, DNA was extracted from 200 μL of the supernatant by utilizing the GENE PREP STAR PI-480 instrument (Kurabo Industries Ltd., Osaka, Japan) according to the manufacturer’s protocol. Amplification of the V3-V4 region of the bacterial 16S rRNA gene through PCR and subsequent DNA sequencing were carried out as previously described [35], using by the Illumina MiSeq instrument (Illumina, San Diego, CA, USA).

Following the removal of sequences that aligned with data from the Genome Reference Consortium human build 38 (GRCh38) and phiX reads from the raw Illumina paired-end reads, the remaining sequences were analyzed using the QIIME2 software package version 2017.10 (https://qiime2.org/). DADA2 [36] was employed to remove potential chimeric sequences, followed by trimming 30 and 90 bases of the 3′ region of the forward and reverse reads, respectively. Taxonomical classification was conducted by utilizing the naive Bayes classifier trained on Greengenes13.8 with a 99% threshold of full-length sequence operational taxonomic units. Alpha diversities were calculated using QIIME2 software. We used R software (ver. 3.6.0) for principal coordinate analysis (PCoA) based on Jensen–Shannon distance (JSD) and partitioning around medoid (PAM) clustering [37]. The optimal number of clusters was estimated by utilizing the Calinski–Harabasz (CH) index.

### 2.6. Quantification of Bifidobacterium Species by Real-Time PCR

Fecal DNA for real-time PCR was extracted as previously described [38]. In brief, 200 μL of fecal sample in GTC buffer was lysed using a Precellys Evolution (Bertin Instruments, FRA) system, and DNA was extracted using the GENE PREP STAR PI-480 instrument (Kurabo Industries Ltd., Osaka, Japan) in accordance with the manufacturer’s protocol. Real-time PCR was conducted using an ABI PRISM 7500 fast real-time PCR system (Thermo Fisher Scientific, Waltham, MA, USA) with TB Green Premix Ex Taq™ Tli RNaseH Plus (TaKaRa Bio Inc., Shiga, Japan) in accordance with the manufacturer’s protocol. Quantification of each Bifidobacteria species was performed using the following primers (Table S1). Primers and amplification methods used were determined based on prior studies [39,40,41]. Bacterial copy numbers were determined by utilizing bacterial solutions with established counts as a standard. Duplicate assays were performed for all samples.

### 2.7. Fecal pH, Short-Chain Fatty Acids (SCFAs) and Biomarker Analysis

Fecal pH was measured with an electrode-fitted pH meter LAQUAtwinB-712 (HORIBA, Kyoto, Japan) after suspending fecal samples in Milli-Q water and sterilizing at 85 °C for 15 min for sterilization of fecal samples.

Fecal SCFAs (acetate, propionate, n-butyrate, iso-butyrate, n-valerate, iso-valerate, n-caproic acid) in ethyl acetate extract were determined by gas chromatography system GC-FID (7890B, Agilent Technologies, Santa Clara, CA, USA) and a DB-WAXetr column (30 m, 0.25 mm id, 0.25 μm film thickness, 1.2 mL/min) as described previously [42].

Commercially available ELISA kits were used to determine fecal secretory immunoglobulin A (sIgA: Human IgA ELISA Core Kit, LABISKOMA, Seoul, Republic of Korea) and calprotectin (IDK^®^ Calprotectin MRP 8/14 ELISA kit, Immundiagnostik AG, Bensheim, Germany).

### 2.8. Sample Size

In a previous study on Japanese infants [15], the relative abundance of bifidobacteria in feces at 1 month of age was 55.40 ± 40%. Assuming the relative abundance of bifidobacteria would increase to 75% by intake of test food, to detect an intergroup difference with 80% power and α = 0.05 and a 15% attrition rate, 75 infants were needed in each group.

### 2.9. Primary and Secondary Outcomes

Evaluation of the primary outcome involved determining whether *B. infantis* M-63 supplementation could increase the relative abundance of Bifidobacteriaceae, the proportion of infants with Bifidobacteriaceae-predominant microbiota, and the fecal copy number of Bifidobacteriaceae in healthy term infants. Secondary outcomes included the effect of *B. infantis* M-63 on the abundance of intestinal bacteria and diversity, GI tolerability (stool frequency and consistency, number of regurgitation and vomiting after feeding), duration and episodes of crying, episodes of fever (≥38 °C), hospital visit, fecal pH, SCFAs, sIgA, and calprotectin.

### 2.10. Statistics

Intergroup differences in the microbiota at the amplicon sequence variant (ASV) level were analyzed by ALDEx2 [43]. A Q-value < 0.05 was considered significant. To assess the variation in microbiota composition explained by each factor, a permutational multivariate analysis of variance (PERMANOVA) test for JSD was used for multivariate analysis. For the relative abundance of Bifidobacteria, a normal log transformation was performed for each time point and for each test food group, and the mean ± standard error of the mean (SEM) was calculated. When the assumption of a normal distribution was not ruled out, an analysis of covariance (ANCOVA) was performed with the values at each time point as the objective variable, the test food group as the explanatory variable, and the baseline values as covariates. The changes from the baseline value were calculated for each time point, and a paired t test was performed. When the assumption of a normal distribution was not verified, the change from the baseline values was calculated for each time point and compared between the groups using the Wilcoxon rank sum test. To identify changes from baseline values, a Wilcoxon signed ranks test was performed. The same analysis was performed for the absolute number of bifidobacteria per gram of feces (copy number), occupancy of each bacterial species and diversity index in gut microbiota analysis, and physical and chemical analysis of stool (pH, amount of short-chain fatty acids, IgA, and calprotectin, pH). For frequency of defecation and fecal characteristics, the average number of defecations (times/day) and average fecal characteristics (score/times) were calculated for each time point and analyzed in the same way. The percentage of infants whose most prevalent bacterial species was *Bifidobacterium* was calculated for each time point, and Fisher’s exact test was performed. The breastfeeding rate was defined as the number of times breastfeeding occurred relative to the number of feedings. Correlations between the breastfeeding rate and the relative abundance of *Bifidobacterium* were analyzed by calculating Spearman’s rank correlation coefficients. For the health condition of the infants, duration and frequency of crying, frequency of regurgitation and vomiting, frequency of fever, and hospital visits, the mean values per week were calculated for each time point, and a Wilcoxon rank sum test was performed.

For the primary endpoint, subgroups were analyzed according to the following factors affecting gut microbiota: (1) mode of delivery (vaginal delivery, cesarean section); (2) whether antibiotics were administered to the mother during delivery; and (3) mode of nutrition (exclusively breastfeeding, mixed feeding, exclusively formula feeding). For height, weight, and head circumference of children, the mean ± SE was calculated for each time point, and Student’s *t* test was performed. Adverse events and side effects were tested for incidence (person/person) using Fisher’s exact test in the ITT population of patients that consumed the study foods. Statistical analysis software was IBM SPSS Statistics (version 28.0), and a *p* value of < 0.05 was adopted to indicate a significant difference.

## 3. Results

### 3.1. Infant and Maternal Characteristics

**Figure 1:**
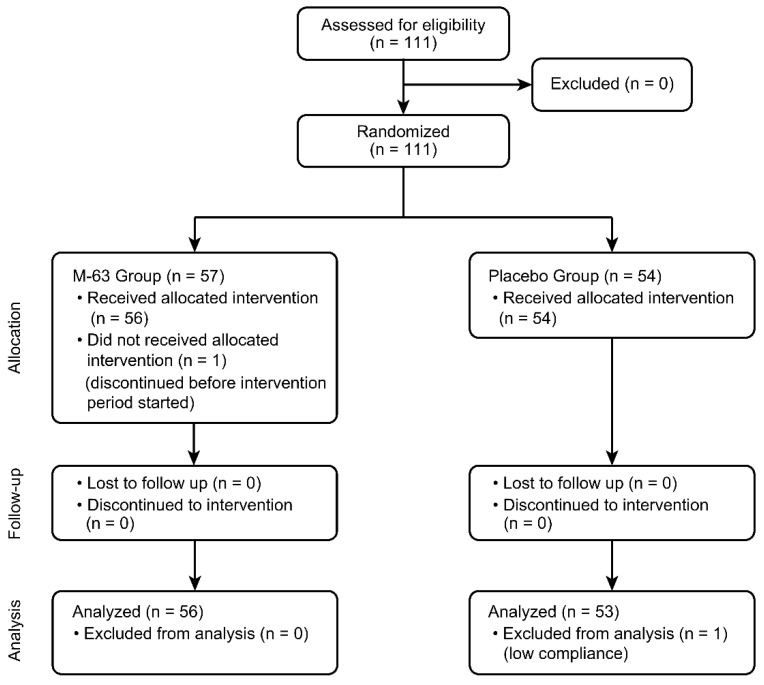
CONSORT flow diagram showing participant flow through the trial.

Figure 1 shows the process from subject enrollment to analysis: 111 subjects were enrolled, and after randomization (54 in the placebo group and 57 in the M-63 group), one subject in the M-63 group withdrew consent before consuming the test food, and one subject in the placebo group was excluded from the analysis due to noncompliance involving intake of less than 50% of the test food. The analysis was performed in the per protocol set (PPS) population (53 patients in the placebo group and 56 patients in the M-63 group). No infant received antibiotics or oral probiotics other than the test food, although one infant used antibacterial eye drops due to discharge from the eyes during the study period.

The subject background of the newborns and mothers is shown in Table 1. There were no significant differences between groups in the number of weeks of gestation, mode of delivery (vaginal delivery, cesarean section), sex, height at birth, weight, head circumference, APGAR score (5 min after birth), and intake rate of test foods among the newborns. There were also no significant differences between the two groups in maternal age, number of deliveries (first-time mothers and term mothers), prepregnancy body mass index (BMI, kg/m^2^), weight gain during pregnancy, number of women taking antibiotics at delivery, history of allergies, and smoking habits.

### 3.2. Infant Feeding

The intake rate of the test foods was 92.4 ± 1.4% for the placebo group and 94.4 ± 0.8% for the M-63 group with no significant difference between groups (*p* = 0.5082). Table S2 shows the number of feedings and the percentage of breastfeeding in each group at 1 week, 1 month, and 3 months of age (calculated from the number of breastfeeds/number of feedings per day). The percentage of breastfeeding increased in both groups at 1 month and 3 months of age with no significant differences between the groups. At 1 month and 3 months of age, 27.5% and 52.3% of the infants were exclusively breastfed, 70.6% and 41.3% were mixed fed, and very few were exclusively formula fed: one case at 1 week, two cases at 1 month, and seven cases at 3 months of age. Therefore, when subgroup analysis was performed, depending on the infant’s feeding status, the analysis was performed in the groups of either exclusive breastfeeding or mixed feeding with exclusive formula feeding.

There were 14 cases of mothers taking antibiotics during the lactation period (6 in the placebo group and 8 in the M-63 group), and there was no significant difference between the two groups. The reasons for taking the drugs were mastitis (three cases), cold (two cases), treatment of cesarean section wounds (one case), uterine restoration failure (one case), treatment of placental abruption (one case), asthma (one case), sinusitis (one case), cystitis (one case), dental treatment (one case), stye (one case), and unknown (one case).

### 3.3. Microbiota Analysis

**Figure 2:**
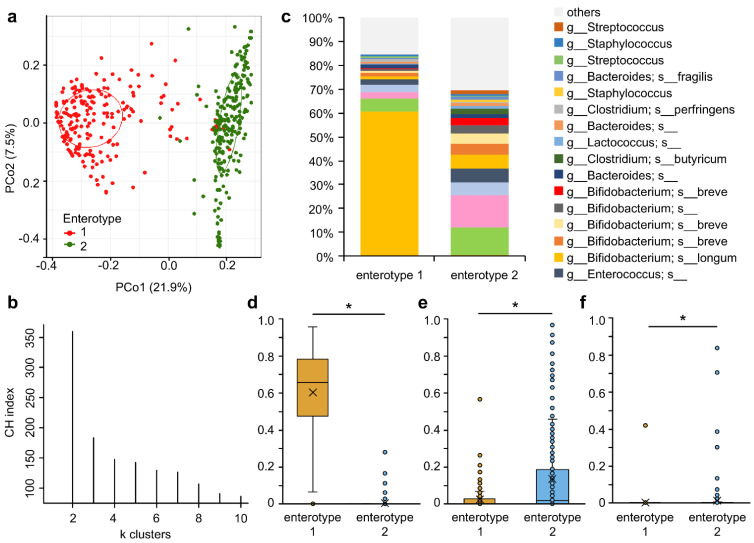
Enterotype of subjects during the intervention. (**a**) Principal coordinate analysis (PCoA) as seen through the first two principal coordinates (PCo1 and PCo2) based on Jensen–Shannon distance (JSD) calculated by the fecal microbiota composition at the ASV level; (**b**) PAMs clustering with JSD. The estimated suitability of the cluster number Calinski–Harabasz index; (**c**) top 20 ASVs in the fecal microbiota composition in each enterotype; (**d**) relative abundance of ASVs annotated as the *B. longum* group, including *B. longum* subsp. *infantis* and *B. longum* subsp. *longum*; (**e**) the relative abundance of ASVs annotated as *Enterobacteriaceae*; (**f**) the relative abundance of ASV annotated as *Lactococcus*. Asterisks (*) indicate *q* < 0.05 calculated by ALDEx2.

**Figure 3:**
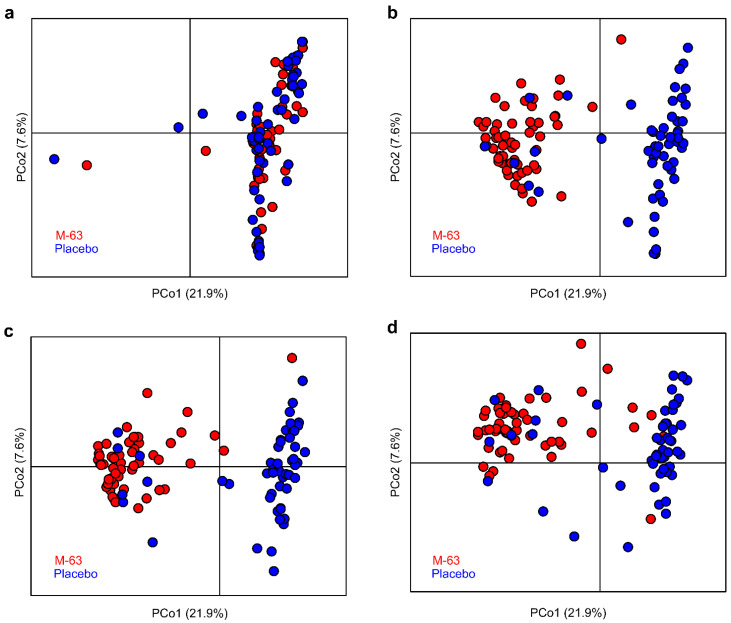
Dynamics of enterotype in each group during the intervention. PCoA of fecal microbiota in each group based on JSD calculated by the fecal microbiota composition at the ASV level. (**a**) Before ingestion; (**b**) 1 week after ingestion; (**c**) 1 month; (**d**) 3 months of age. Blue and red symbols indicate subjects in the placebo and M-63 groups, respectively.

We then investigated the fecal microbiota of all samples during the intervention. PCoA data clearly showed two enterotypes (Figure 2a,b) enriched in *Bifidobacterium* (enterotype 1) and several taxa, such as Enterobacteriaceae (enterotype 2, Figure 2c–f). Within a week of birth, 101 of 106 subjects had enterotype 2 microbiota (Figure 3a). However, the enterotype of all subjects in the M-63 group except one transitioned to the *Bifidobacterium*-dominated enterotype (enterotype 1) after 1 week of administration of *B. infantis* M-63, while the enterotype of most subjects in the placebo group was stable in enterotype 2 (Figure 3). This polarization was maintained until the end of the intervention.

### 3.4. Bifidobacterial Colonization

The relative abundance of *Bifidobacterium* and the percentage of infants for whom *Bifidobacterium* spp. was the most dominant bacterial genus are shown in Table 2. There was no significant difference between the two groups in the relative abundance of *Bifidobacterium* before intake of the test food, and the relative abundance of *Bifidobacterium* increased with the age of the infants (1 week, 1 month, and 3 months of age), and a significant increase was observed in the M-63 group over the placebo group. The percentage of infants in the M-63 group with *Bifidobacterium* as the most dominant genus was also significantly higher than that of the placebo group at the first week of intake and at one month of age. This trend was also observed in all subgroups, regardless of the delivery method (vaginal or cesarean section), regardless of the mode of nutrition in the infants or whether antibiotics were used by the mothers at delivery.

The relative abundance of bifidobacteria in the placebo group was significantly lower in infants born to mothers who received antibiotics at delivery than in those whose mothers did not receive antibiotics before intake and was also lower in the placebo group at 1 week (Table S3). There was no significant difference in the relative abundance of bifidobacteria between cesarean section and vaginal delivery infants within the population whose mothers received antibiotics in the placebo group, and no association was found between mode of delivery and the relative abundance of bifidobacteria. Even in the case of vaginal delivery, bifidobacteria were found to be significantly lower in the antibiotic-treated group than in the antibiotic-untreated group.

### 3.5. Bacterial Species of Colonized Bifidobacteria

The abundance and detection rate of the genus *Bifidobacterium* and each *Bifidobacterium* species at 1 month after birth, measured by quantitative PCR, are shown in Table 3. There were no significant differences in the abundance and detection rate of the genus *Bifidobacterium* and each *Bifidobacterium* species before the ingestion point. However, the abundance and detection rate of the genera *Bifidobacterium* and *B. infantis* in the M-63 group were significantly higher than those in the placebo group. In short, the results of quantitative PCR at 1 month of age showed that most of the *Bifidobacterium* species in the M-63 group were *B. infantis*, whereas *B. infantis* was barely detected in the placebo group and *B. breve* and *B. longum* subsp. *longum* (*B. longum*) were detected in the placebo group. These results indicate that *B. infantis* was the main *Bifidobacterium* species that colonized the intestines of the infants in the M-63 group, while *B. breve* and *B. longum* were the main species in the placebo group.

### 3.6. Correlation between Breastfeeding and Bifidobacterial Occupancy

**Figure 4:**
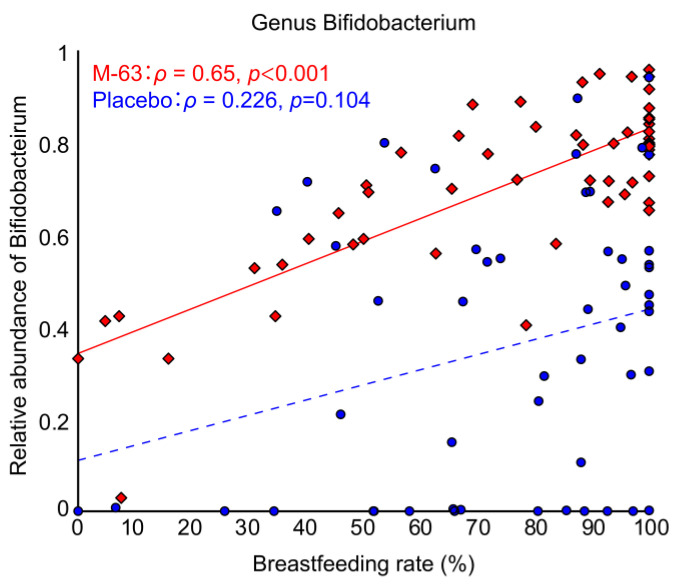
Correlation between the breastfeeding rate and the relative abundance of *Bifidobacterium* at 1 month of age. Red squares (◆) indicate the M-63 group, and blue circles (●) indicate the placebo group. Spearman’s correlation coefficients and the corresponding *p* values are shown.

We analyzed the correlation at 1 month of age between the breastfeeding rate and the relative abundance of bifidobacteria (Figure 4). A significant positive correlation was seen between the breastfeeding rate and the relative abundance of *Bifidobacterium* in the M-63 group. However, there was no significant positive correlation between them in the placebo group. Although the breastfeeding rate was more than 80%, some infants did not have increased bifidobacteria in the gut in the placebo group.

### 3.7. Gut Fermentation Patterns and Immunologic Parameters in Stools

The pH, amount of short-chain fatty acid, IgA, and calprotectin in the stools before intake and one month after birth are shown in Table 4. In the M-63 group, the pH in the stools was significantly lower and the amount of acetic acid in the stools increased at 1 month of age compared to the placebo group. There was no difference between the two groups in terms of calprotectin in the feces, but IgA levels in the feces were significantly higher in the M-63 group than in the placebo group at one month of age.

### 3.8. GI Tolerability and Health Condition of Infants

**Figure 5:**
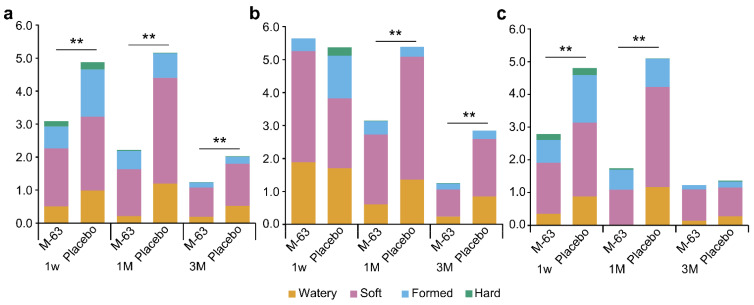
Number of infant stools and consistency. (**a**) PPS population; (**b**) exclusively breastfed infants; (**c**) mixed-fed infants and exclusively formula-fed infants. ** *p* < 0.01 vs. placebo group (Wilcoxon rank sum test).

The number of defecations per day and fecal characteristics of the infants are shown in Figure 5. The placebo group showed a decrease in defecation frequency from 1 to 3 months of age, whereas the M-63 group showed a gradual decrease in defecation frequency from 1 week to 3 months of age, with a significant decrease in the M-63 group compared to the placebo group”. “Watery” stools and “soft” stools were significantly decreased in the M-63 group as compared to the placebo group at 1 month of age (Figure 5a). Stratified analysis by type of nutrition is shown in Figure 5b,c. In the exclusively breastfed infants, defecation frequency was lower in the M-63 group than in the placebo group at 1 and 3 months of age (Figure 5b), and in the mixed-fed infants and exclusively formula-fed infants, defecation frequency was lower in the M-63 group than in the placebo group at 1 week and 1 month of age (Figure 5c). These results indicate that M-63 modulates the defecation function of infants regardless of the type of nutrition.

The number of times (times/day) and average duration (minutes/time) that infants cried for more than 30 min are shown in Table S4. Neither group included infants who cried for an average of more than 3 h per day or who were suspected of having so-called “colic”, and there were no significant differences between the two groups.

The number of times and number of infants with regurgitation and vomiting of milk are shown in Table S5. There were significantly fewer infants in the M-63 group than in the placebo group who had regurgitation of milk at 1 week after intake. Both regurgitation and vomiting of milk were mild and not pathological.

The growth of the infant’s height, weight, and head circumference at 1 and 3 months after birth was favorable and comparable in both groups (Table S6).

### 3.9. Adverse Events

Adverse events during the study period are shown in Table S7. No adverse events attributable to bifidobacteria administration were identified, and the incidence of respiratory, gastrointestinal, and skin symptoms did not differ between the two groups.

## 4. Discussion

In this study, *B. infantis* M-63 was administered as a single species probiotic at a dose of 1 billion/day to healthy full-term infants up to 3 months of age to investigate its effects on gut microbiota formation, intestinal environment, gastrointestinal function and fecal immune parameters. Administration of *B. infantis* M-63 was associated with a decreased frequency of defecation and watery stools (Figure 5), suggesting that *B. infantis* M-63 may modulate gastrointestinal function in infants. Ingestion of *B. infantis* M-63 promoted the formation of *Bifidobacterium*-dominant gut microbiota within one week (Figure 2 and Figure 3), and this effect was confirmed even in infants with low postnatal bifidobacterial occupancy, such as cesarean section infants and newborn infants born to mothers who had taken antibiotics during delivery (Table 2). In the M-63 group, the relative abundance of *Bifidobacterium* was higher as the frequency of breast milk intake increased (Figure 4), and the main *Bifidobacterium* species that colonized the infants was *B. infantis* (Table 3), indicating that *B. infantis* is compatible with breast milk. Administration of *B. infantis* M-63 was shown to decrease stool pH by increasing the amount of acetic acid in the stool and to increase IgA in the stool (Table 4), which plays an important role in mucosal immunity. No serious adverse events were observed in neonates that could be attributed to *B. infantis* M-63 administration (Table S7). These results indicate that administration of *B. infantis* M-63 at 1 billion/day to healthy full-term neonates has a beneficial effect on the health maintenance of infants by promoting the formation of Bifidobacterium-dominant gut microbiota.

The frequency of defecation during the intervention decreased over the first 3 months of life in both groups (Figure 5). This is consistent with reports that defecation frequency decreases physiologically during the neonatal period. This may be due to the maturation of the digestive and absorptive capacity of nutrients and water as the intestinal tract grows [44,45,46,47]. The frequency of defecation was significantly decreased in the M-63 group compared to the placebo group, especially the decrease in watery stools, which was remarkable. A clinical trial using *B. infantis* EVC001 in neonates showed a similar effect [48]. The authors speculate that *B. infantis* consumption promoted the maturation of the intestinal mucosa, based on the fact that *Bifidobacterium* enhances the barrier function of the intestinal tract [6] and that *B. infantis* increases the expression of mRNA for tight junction proteins in intestinal epithelial cells [49]. On the other hand, in the present study, a modulatory effect of *B. infantis* M-63 administration on defecation frequency and fecal characteristics was observed from 1 week after ingestion. Since this effect was observed immediately after ingestion, it is possible that administration of M-63 directly regulates intestinal functions via a decrease in intestinal pH, etc., rather than promoting the development of intestinal functions. Considering these results and reports, it is possible that administration of M-63 to neonates regulates intestinal functions through direct modulatory effects via a decrease in intestinal pH, etc., in addition to promoting the development of intestinal functions.

The gut microbiota of breastfed infants was reported to be dominated by *Bifidobacterium* until the cessation of breastfeeding [50,51]. Even though almost all infants were breastfed or mixed-fed at 1 week after intake (Table S2), in the placebo group, the percentage of infants where *Bifidobacterium* was the most dominant genus was 41.5%, and this proportion was still only 51% at 1 month of age (Table 2). Furthermore, *B. infantis* was not detected in any except four of the placebo group infants by 1 month of age (Table 3). On the other hand, *B. infantis* was not detected before intake in the M-63 group but was detected in 54 of 56 infants at 1 month of age (Table 3), and the proportion of infants with *Bifidobacterium* as the most prevalent species increased sharply to 94.6% (Table 2). The majority of bifidobacteria that caused the increased with intake of M-63 were *B. infantis*, while the amounts of *B. longum*, *B. bifidum*, and *B. breve*, which were present before the intervention, did not change significantly (Table 3). It has been reported that *B. infantis* is undetectable in many adults and is detected in infants after 2 months of age, indicating the possibility of horizontal transmission from the skin or environment rather than vertical transmission from mother to infant during delivery [52]. In the present study, *B. infantis* was detected in most of the infants in the M-63 group at 1 week after intake (Table 3), i.e., less than 1 month after birth, and we speculate that the *B. infantis* M-63 administered soon after birth colonized the intestine and formed a bifidobacteria-dominant gut microbiota earlier than in the nonintervention population.

In the M-63 group, there was a significant positive correlation between the breastfeeding rate and the relative abundance of *Bifidobacterium* (Figure 4), and the main *Bifidobacterium* species that colonized the infants in the M-63 group was *B. infantis* (Table 3). These results suggest that breast milk intake increases bifidobacteria in the infant gut, especially *B. infantis* among the bifidobacteria species. On the other hand, no correlation was found between the proportion of breastfeeding and bifidobacteria occupancy in the placebo group (Figure 4). A certain number of infants in the placebo group showed no bifidobacterial colonization even though they were breastfed a higher proportion of the time. These results suggest that there are cases in which Bifidobacteria do not colonize even with breastfeeding and that supplementation with *B. infantis* in the early postnatal period and either breastfeeding or supplementation of alternative breast milk components may be necessary to establish a high percentage of Bifidobacteria.

Maternal antimicrobial use at delivery has been reported to have a stronger effect than the mode of delivery on the gut microbiota, in particularly colonization of bifidobacteria [13,53]. In the present study, neonates of mothers who used antimicrobials at delivery had lower bifidobacterial occupancy within the first 7 days of life, whereas mode of delivery had no clear effect on neonatal bifidobacterial abundance (Table S3), suggesting that the establishment of bifidobacteria in the infant intestinal tract is more influenced by the mother’s use of antimicrobials during delivery than by the mode of delivery. Administration of *B. infantis* M-63 was found to be effective in increasing bifidobacterial abundance, which was low due to the use of antimicrobials at delivery, by 1 week after administration (Table 2).

Evaluation of intestinal metabolites at 1 month of intervention showed a significant increase in acetic acid in stools and a decrease in n-butanoic acid in the M-63 group compared to the placebo group (Table 4). This is consistent with reports of other bifidobacteria administered neonatally and changes in short-chain fatty acids in the gut [54]. Acetic acid and butyric acid are beneficial short-chain fatty acids and have been reported to change composition gradually in the infant’s intestine [55]. Butyrate is an important short-chain fatty acid that is a major source of energy for intestinal epithelial cells, but it is not a major source during lactation as it has been reported to increase with intestinal Clostridiales occupancy after lactation cessation [55]. Patients in the M-63 group showed a significant decrease in fecal pH after 1 month of intervention compared to the placebo group (Table 4), and there was a significant negative correlation between the number of bifidobacteria in the feces and pH (*ρ* = −0.716, *p* < 0.01. A review of studies of infant fecal pH and gut microbiota over the past 100 years indicates an increase in infant fecal pH and a concomitant decrease in bifidobacteria, a major component of infant gut commensal bacteria [56]. More interestingly, in relation to *B. infantis*, infants lacking *B. infantis* have been reported to have significantly higher fecal pH, higher levels of potential pathogens and mucus-eating bacteria in their intestinal flora, and signs of chronic enteritis, suggesting that acidification in the gut by *B. infantis* may contribute to the suppression of harmful bacteria associated with the induction of inflammation [56]. The study confirmed that consumption of *B. infantis* M-63 has the effect of increasing acetic acid concentration and lowering pH in the gut, which may have beneficial effects on the health of newborns.

Ingestion of *B. infantis* M-63 resulted in increased IgA in stool one month after intervention (Table 4). IgA is a mucosal immunoglobulin that predominates in mucosal tissues such as the intestinal tract and plays a crucial role in protection against antigens, toxins, and potential pathogens. It has been reported that IgA is increased in breast-fed infants due to the supply of IgA from breast milk. Although the IgA concentration in breast milk was not measured in this study, the breast milk intake rate was similar to that of the placebo group (Table S2), and no significant correlation was observed between the breast milk intake rate and IgA concentration in placebo group (*ρ* = −0.005, *p* = 0.973). A trend toward increased IgA was observed in the M-63 group even in the mixed-fed infants and exclusively formula-fed infants (M-63: 1832 ± 249 µg/g, placebo: 1352 ± 187 µg/g; *p* = 0.299), suggesting that the increase in *B. infantis* may have promoted IgA secretion from the intestinal immune tissues. Similar to our findings, bifidobacteria in the intestine, especially *B. infantis* and *B. breve*, have been demonstrated to increase fecal sIgA and anti-poliovirus-specific IgA in healthy full-term infants. Acetic acid has been reported to promote IgA secretion in the intestinal tract by regulating the IgA class switch of B cells by intestinal dendritic cells via GPR43, thereby maintaining host-intestinal bacterial symbiosis and exhibiting anti-inflammatory effects [57]. The effect of *B. infantis* M-63 on the host immune response requires detailed analysis, but ingestion of *B. infantis* M-63 may promote IgA secretion into the intestinal tract by increasing acetic acid in the intestinal tract.

Frese et al. [58] reported that administration of *B. infantis* EVC001 at a dose of 18 billion CFU/day to full-term infants significantly increased bifidobacteria in the intestine. In the present study, administration of a lower dose of *B. infantis* M-63 at 1 billion CFU/day to full-term infants significantly increased *B. infantis* in the gut (Table 2), and the relative abundance of bifidobacteria, most of which were presumed to be *B. infantis* species in the M-63 group, correlated with the proportion of breastfeeding. *B. infantis* is known to have a generally high capacity to utilize HMOs [59], and *B. infantis* M-63 has also been reported to have a high capacity to utilize the major HMOs in human breast milk [23,24]. It is likely that *B. infantis* M-63 preferentially utilizes HMOs in the gut of breast-fed infants, allowing it to grow efficiently and form a *Bifidobacterium*-dominated microbiota even at low doses.

Mothers recorded the number of episodes and duration of crying for more than 30 min and the number of instances of regurgitation and vomiting milk in their diaries for the seven days prior to 1 week, 1 month, and 3 months of age. There was no difference between the groups in the number of episodes and duration of crying for more than 30 min (Table S4). Regarding regurgitation and vomiting milk, the frequency of regurgitation tended to be higher in the M-63 group at the age of 3 months, but they were not pathological or serious events (Table S5). There were no differences between both groups in other adverse events recorded in the logs during the study period (Table S7), and no adverse events were identified that could be attributed to the consumption of the test food. There was also no difference in the growth of the infants up to 3 months of age (Table S6). These results indicate that ingestion of *B. infantis* M-63 is safe and well tolerated in neonates.

Potential limitations of this study are that it was conducted at a single center in Japan and that we were unable to evaluate the effects of the composition of breast milk on the infant’s gut microbiota and immune indices. However, the effects of breast milk should have been limited to some extent, as confirmed by the fact that breast milk frequency was studied and was comparable in both feeding groups. To obtain a comprehensive understanding of early bifidobacterial intervention and its beneficial effects on healthy full-term infants, a detailed analysis of the association between gut microbiota and clinical benefit is needed, and the results of this analysis will be reported as a separate study. Further studies, including long-term follow-ups, are also needed to determine the impact of early bifidobacterial intervention on the health of growing infants.

## 5. Conclusions

In conclusion, supplementation with *B. infantis* M-63 in healthy term infants was well tolerated and beneficially modulated the infant gut microbiota toward higher *Bifidobacterium* levels, accompanied by softer stool consistency. *B. infantis* M-63 enhances the secretion of intestinal acetic acid and sIgA, providing beneficial effects on digestive function.

## Figures and Tables

**Table 1:** Subject background of infants and mothers.

		Placebo (n = 53)	M-63 (n = 56)	*p* Value
Infant				
	Gestational age, weeks	39.0 ± 0.2	39.1 ± 0.2	0.704 ^a^
	Singleton, n (%)	53 (100)	56 (100)	-
	Sex, n male/female (%)	24/29 (45.3/54.7)	28/28 (50.0/50.0)	0.702 ^b^
	Birth weight, g	3037.5 ± 43.6	3058.7 ± 35.4	0.706 ^a^
	Birth height, cm	49.2 ± 0.2	49.5 ± 0.2	0.319 ^a^
	Birth head circumference, cm	33.4 ± 0.2	33.5 ± 0.2	0.496 ^a^
	Cesarean births, n (%)	8 (15.1)	11 (19.6)	0.618 ^b^
	APGAR score (5 min after birth)	8.96 ± 0.05	9.07 ± 0.04	0.087 ^a^
Maternal				
	Maternal age, years	31.8 ± 0.7	31.3 ± 0.6	0.534 ^a^
	Multiparous woman, n (%)	36 (68.0)	32 (57.1)	0.323 ^b^
	Prepregnancy body mass index (BMI)	21.0 ± 0.4	21.8 ± 0.4	0.119 ^a^
	Pregnancy weight gain, kg	9.9 ± 0.4	9.7 ± 0.5	0.759 ^a^
	Antibiotics during labor, n (%)	30 (56.6)	30 (53.6)	0.848 ^b^
	Pregnancy smoking habits, n (%)	3 (5.7)	3 (5.4)	1.00 ^b^

Values are represented as the mean ± SEM, ^a^ Student’s *t* test, ^b^ Fisher’s exact test.

**Table 2:** The relative abundance of *Bifidobacterium* and percentage of infants in which *Bifidobacterium* was the most dominant genus.

		Relative Abundance of *Bifidobacterium (%)*			Infants Where *Bifidobacterium* Is The Most Dominant Genus, n (%)		
		Placebo	M-63	*p* Value ^a^	Placebo	M-63	*p* Value ^b^
PPS population							
	Before ingestion	18.7 ± 4.0	17.1 ± 3.4	0.531	16 (30.2)	15 (26.8)	0.832
	1 week after ingestion	28.3 ± 3.8	63.8 ± 2.2	<0.001	22 (41.5)	53 (94.6)	<0.001
	1 month of age	35.8 ± 4.1	71.0 ± 2.5	<0.001	27 (51.0)	53 (94.6)	<0.001
	3 months of age	44.3 ± 3.3	64.5 ± 3.0	<0.001	36 (68.0)	47 (83.9)	0.072
Vaginal delivery							
	Before ingestion	20.2 ± 4.2	18.4 ± 3.8	0.669	15 (33.3)	13 (29.0)	0.82
	1 week after ingestion	29.8 ± 4.2	62.1 ± 2.5	<0.001	20 (44.4)	42 (93.3)	<0.001
	1 month of age	37.6 ± 4.4	69.4 ± 2.9	<0.001	24 (53.3)	42 (93.3)	<0.001
	3 months of age	43.5 ± 3.7	63.4 ± 3.4	<0.001	29 (64.4)	38 (84.4)	0.052
Cesarean section							
	Before ingestion	10.3 ± 10.2	11.8 ± 8.0	0.560	1 (12.5)	2 (18.2)	1.00
	1 week after ingestion	20.4 ± 8.3	70.7 ± 4.2	<0.001	2 (25.0)	11 (100.0)	0.001
	1 month of age	26.0 ± 12.1	77.7 ± 3.5	0.004	3 (37.5)	11 (100.0)	0.005
	3 months of age	48.5 ± 9.8	69.0 ± 6.2	0.099	7 (87.5)	9 (81.8)	1.00
Not using antibiotics during labor							
	Before ingestion	27.5 ± 5.5	27.3 ± 5.4	0.992	11 (47.8)	11 (42.3)	0.778
	1 week after ingestion	37.8 ± 5.9	64.4 ± 3.8	0.001	14 (60.9)	25 (96.2)	0.003
	1 month of age	40.5 ± 5.7	67.6 ± 4.3	<0.001	12 (52.2)	23 (88.5)	0.010
	3 months of age	42.7 ± 5.1	62.4 ± 4.8	0.006	14 (60.9)	21 (80.8)	0.205
Using antibiotics during labor							
	Before ingestion	12.0 ± 5.2	8.3 ± 3.6	0.324	5 (16.7)	4 (13.3)	1.00
	1 week after ingestion	21.1 ± 4.6	63.3 ± 2.5	<0.001	8 (26.7)	28 (93.3)	<0.001
	1 month of age	32.2 ± 5.2	74.0 ± 2.6	<0.001	15 (50.0)	30 (100.0)	<0.001
	3 months of age	44.1 ± 4.5	66.4 ± 3.8	0.001	22 (73.3)	26 (86.7)	0.333
Breast-fed infants							
	1 week after ingestion	36.1 ± 10.3	68.0 ± 4.4	0.022	4 (57.1)	6 (100.0)	0.192
	1 month of age	45.5 ± 8.5	81.3 ± 1.8	<0.001	7 (63.6)	19 (100.0)	0.012
	3 months of age	49.5 ± 5.6	72.9 ± 2.7	<0.001	18 (75.0)	32 (97.0)	0.034
Mixed-fed infants and formula-fed infants							
	1 week after ingestion	27.2 ± 4.1	63.3 ± 2.4	<0.001	18 (39.1)	47 (94.0)	<0.001
	1 month of age	33.3 ± 4.7	65.7 ± 3.3	<0.001	20 (47.6)	34 (91.9)	<0.001
	3 months of age	38.5 ± 3.9	52.5 ± 5.3	0.052	18 (62.1)	15 (65.2)	1.00

Values are represented as the mean ± SEM, *p* < 0.05 indicates statistical significance, ^a^ Wilcoxon rank sum test, ^b^ Fisher’s exact test.

**Table 3:** Abundance and detection rate of *Bifidobacterium* species.

		Abundance (Log_10_ CFU/g Feces)			Number of Infants Where Each *Bifidobacterium* Was Detected (Detection Rate, %)		
		Placebo	M-63	*p* Value ^a^	Placebo	M-63	*p* Value ^b^
Genus *Bifidobacterium*							
	Before ingestion	7.57 ± 0.27	7.85 ± 0.27	0.487	24 (45.3)	29 (51.2)	0.567
	1 month of age	9.10 ± 0.29	10.73 ± 0.11	<0.001	38 (71.7)	55 (98.2)	<0.001
*Bifidobacterium bifidum*							
	Before ingestion	6.20 ± 0.12	6.25 ± 0.11	0.374	3 (5.7)	6 (10.7)	0.490
	1 month of age	6.42 ± 0.17	6.52 ± 0.17	0.425	6 (11.3)	10 (17.9)	0.421
*Bifidobacterium breve*							
	Before ingestion	6.50 ± 0.16	6.68 ± 0.18	0.543	10 (18.9)	13 (23.2)	0.643
	1 month of age	7.36 ± 0.25	7.07 ± 0.19	0.523	21 (39.6)	24 (42.9)	0.846
*Bifidobacterium longum*							
	Before ingestion	6.85 ± 0.19	7.19 ± 0.24	0.192	15 (28.3)	21 (37.5)	0.318
	1 month of age	7.39 ± 0.25	7.22 ± 0.20	0.735	24 (45.3)	27 (48.2)	0.848
*Bifidobacterium infantis*							
	Before ingestion	6.15 ± 0.11	6.00 ± 0.00	0.144	2 (3.8)	0 (0)	0.234
	1 month of age	6.35 ± 0.17	10.36 ± 0.13	<0.001	4 (7.6)	54 (96.4)	<0.001

Values are represented as the mean ± SEM, *p* < 0.05 indicates statistical significance, ^a^ Wilcoxon rank sum test, ^b^ Fisher’s exact test.

**Table 4:** pH, amount of short-chain fatty acid, IgA, and calprotectin in the stools.

			Placebo	M-63	*p* Value ^1^
pH					
		Before ingestion	5.92 ± 0.08	6.05 ± 0.08	0.209
		1 month of age	6.05 ± 0.11	5.53 ± 0.07	<0.001
Short-chain fatty acids (µmol/g feces)					
	acetic acid				
		Before ingestion	16.09 ± 1.49	15.45 ± 1.16	0.884
		1 month of age	22.01 ± 1.62	30.0 ± 1.44	<0.001
	propionic acid				
		Before ingestion	0.54 ± 0.26	0.64 ± 0.31	0.644
		1 month of age	2.32 ± 0.66	2.54 ± 0.69	0.404
	n-butanoic acid				
		Before ingestion	0.43 ± 0.36	0.75 ± 0.34	0.105
		1 month of age	0.49 ± 0.17	0.15 ± 0.11	0.027
	iso-butanoic acid				
		Before ingestion	0	0.01 ± 0.01	0.326
		1 month of age	0.06 ± 0.02	0.08 ± 0.04	0.734
	n-valeric acid				
		Before ingestion	0	0	1.00
		1 month of age	0.02 ± 0.02	0.01 ± 0.01	0.931
	iso-valeric acid				
		Before ingestion	0.01 ± 0.01	0.01 ± 0.01	0.989
		1 month of age	0.15 ± 0.09	0.06 ± 0.03	0.795
	n-caproic acid				
		Before ingestion	0.01 ± 0.01	0.01 ± 0.01	0.989
		1 month of age	0.01 ± 0.01	0	0.317
Calprotectin (µg/g feces)					
		Before ingestion	277.06 ± 35.16	346.07 ± 54.22	0.535
		1 month of age	212.54 ± 30.0	257.52 ± 37.46	0.537
IgA (µg/g feces)					
		Before ingestion	1528.85 ± 311.53	1700.63 ± 373.62	0.661
		1 month of age	1393.19 ± 152.91	1971.25 ± 200.90	0.033

All values are represented as the mean ± SEM, *p* < 0.05 indicates statistical significance. ^1^ Wilcoxon rank sum test.

## Data Availability

The data presented in this study can be found in this published article and its Supplementary Information files.

## Supplementary Materials

The following supporting information is available to download at https://www.mdpi.com/article/10.3390/nu15061402/s1: Table S1: PCR primers for the detection of infant intestinal bifidobacteria; Table S2: The number of feedings and percentage of breastfed infants in each group; Table S3: The relative abundance of Bifidobacterium in the placebo group in relation to antibiotics and mode of delivery; Table S4: The number of times and average duration that infants cried for more than 30 min; Table S5: The number of times and number of infants with regurgitation and vomiting of milk; Table S6: Growth of the infant’s height, weight, and head circumference at 1 and 3 months after birth; Table S7: Summary of adverse events during the study period.
