# Supplementary material for: Early Probiotic Supplementation of Healthy Term Infants with Bifidobacterium longum subsp. infantis M-63 Is Safe and Leads to the Development of Bifidobacterium-Predominant Gut Microbiota: A Double-Blind, Placebo-Controlled Trial
Source: Nutrients. 2023 Mar 14;15(6):1402. doi: 10.3390/nu15061402 (PMC10055625; doi:10.3390/nu15061402)
Supplement: Supplementary file 1 [file nutrients-15-01402-s001.zip › nutrients-2250127-supplementary.pdf]

nutrients

Table S1. PCR primers for detection of infant's intestinal bifidobacteria.

Target	Primer	Sequence (5' to 3')	Reference
Genus *Bifidobacterium*	g-Bifid-F	CTCCTGGAAACGGGTGG	[40]
	g-Bifid-R	GGTGTTCTTCCCGATATCTACA	
Bifidobacterium bifidum	BiBIF-1	CCACATGATCGCATGTGATTG	[41]
	BiBIF-2	CCGAAGGCTTGCTCCCAAA	
Bifidobacterium breve	BiBRE-1	CCGGATGCTCCATCACAC	[41]
	BiBRE-2	ACAAAGTGCCTTGCTCCCT	
Bifidobacterium longum. subsp. *longum*	BiLON-1	TTCCAGTTGATCGCATGGTC	[41]
	BiLON-2	GGGAAGCCGTATCTCTACGA	
Bifidobacterium longum. subsp. *infantis*	Sia-266F	GACGAGGAGGAATACAGCAG	[39]
	Sia-676R	CACGAACAGCGAATCATGGATT	

Table S2. The number of feedings and percentage of breastfed infants in each group.

	Placebo	M-63	*p*-Value
Frequency of feeding (times/day)			
1 week after ingestion	10.9 ± 0.3	10.6 ± 0.3	0.469[a]
1 month of age	11.0 ± 0.3	10.9 ± 0.3	0.757[a]
3 months of age	8.3 ± 0.2	8.2 ± 0.2	0.796[a]
Frequency of breast milk feeding (times/day)			
1 week after ingestion	7.3 ± 0.3 (69.0%)	7.0 ± 0.4 (66.6%)	0.495[a]
1 month of age	8.1 ± 0.3 (75.7%)	8.2 ± 0.4 (75.4%)	0.927[a]
3 months of age	6.6 ± 0.3 (79.8%)	6.6 ± 0.4 (78.2%)	0.961[a]
Exclusively breast-fed infants (n, %)			
1 week after ingestion	7 (13.2%)	6 (10.7%)	0.772[b]
1 month of age	11 (20.8%)	19 (34.0%)	0.139[b]
3 months of age	24 (45.3%)	33 (59.0%)	0.182[b]
Exclusively formula-fed infants (n, %)			
1 week after ingestion	0 (0.0%)	1 (1.8%)	—
1 month of age	1 (1.9%)	1 (1.8%)	—
3 months of age	1 (1.9%)	6 (10.7%)	—
Mixed fed infants (n, %)			
1 week after ingestion	46 (86.8%)	49 (87.5%)	1.000[b]
1 month of age	41 (77.4%)	36 (64.3%)	0.147[b]
3 months of age	28 (52.8%)	17 (30.4%)	0.020[b]

Values are represented as the mean ± SEM, $p < 0.05$ are statistically significant, [a] Student's t-test, [b] Fisher's exact test

Table S3. The relative abundance of *Bifidobacterium* in the placebo group in relation to antibiotics and mode of delivery.

	The relative abundance of *Bifidobacterium (%)*			
Antibiotics	Before ingestion	1 week after ingestion	1 month of age	3 months of age
Not using antibiotics during labor (n = 23)	27.5 ± 5.5 *	37.8 ± 5.9 *	40.5 ± 5.7	42.7 ± 5.1
Using antibiotics during labor (n = 30)	8.9 ± 4.3	21.1 ± 4.6	32.3 ± 5.8	44.1 ± 4.5
Mode of delivery				
Vaginal delivery (n = 45)	18.4 ± 3.9	29.8 ± 4.2	37.6 ± 4.4	43.5 ± 3.7
Cesarean section (n = 8)	10.3 ± 10.2	20.4 ± 8.3	26.0 ± 12.1	48.5 ± 9.8
Using antibiotics during labor (n = 30)				
Vaginal delivery (n = 22)	8.4 ± 4.7	21.3 ± 5.6	34.6 ± 6.7	42.5 ± 5.2
Cesarean section (n = 8)	10.3 ± 10.2	20.4 ± 8.3	26.0 ± 12.1	48.5 ± 9.8
Not using antibiotics during labor (n = 23)				
Vaginal delivery (n = 23)	27.5 ± 5.5	37.8 ± 5.9	40.5 ± 5.7	42.7 ± 5.1
Cesarean section (n = 0)	-	-	-	-
Vaginal delivery (n = 45)				
Not using antibiotics during labor (n = 23)	27.5 ± 5.5 *	37.8 ± 5.9 *	40.5 ± 5.7	42.7 ± 5.1
Using antibiotics during labor (n = 22)	8.4 ± 4.7	21.3 ± 5.6	34.6 ± 6.7	42.5 ± 5.2
Cesarean section (n = 8)				
Not using antibiotics during labor (n = 0)	-	-	-	-
Using antibiotics during labor (n = 8)	10.3 ± 10.2	20.4 ± 8.3	26.0 ± 12.1	48.5 ± 9.8

Data for the placebo group only are shown above. All values are represented as the mean ± SEM

* $p<0.05$ are statistically significant between two groups.

Table S4. The number of times and average duration that infants cried for more than 30 minutes.

	Placebo	M-63	*p*-Value
Number of times cried for more than 30 minutes (times/day)			
1 week after ingestion	0.38 ± 0.16	0.29 ± 0.10	0.910 [a]
1 month of age	0.40 ± 0.17	0.32 ± 0.11	0.745 [a]
3 months of age	0.09 ± 0.05	0.14 ± 0.11	0.939 [a]
Average duration times cried for more than 30 minutes (minutes/time)			
1 week after ingestion (n=9, 9)	45.9 ± 4.5	42.6 ± 4.2	0.686 [a]
1 month of age (n=10, 9)	56.3 ± 12.1	61.5 ± 16.3	0.932 [a]
3 months of age (n=3, 2)	36.7 ± 6.7	40.0 ± 10.0	0.739 [a]
Total duration times cried for more than 30 minutes (minutes/week)			
1 week after ingestion (n=9, 9)	125.0 ± 38.9	82.2 ± 18.0	0.562 [a]
1 month of age (n=10, 9)	128.0 ± 38.6	117.8 ± 28.0	0.934 [a]
3 months of age (n=3, 2)	63.3 ± 20.3	165.0 ± 135.0	0.767 [a]
Number of infants that cried for more than 30 minutes			
1 week after ingestion, n (%)	9 (16.1%)	9 (17.0%)	1.000 [b]
1 month of age, n (%)	9 (16.1%)	10 (18.9%)	0.803 [b]
3 months of age, n (%)	3 (5.4%)	3 (5.7%)	1.000 [b]

Values are represented as the mean ± SEM, [a] Wilcoxon rank sum test, [b] Fisher's exact test

Table S5. The number of times and number of infants with regurgitation and vomiting of milk.

	Placebo	M-63	*p*-Value
Number of times of regurgitation (times/day)			
1 week after ingestion	0.57 ± 0.13	0.58 ± 0.13	0.408 [a]
1 month of age	0.69 ± 0.14	0.84 ± 0.20	0.799 [a]
3 months of age	0.38 ± 0.13	0.64 ± 0.19	0.284 [a]
Number of infants with regurgitation (%)			
1 week after ingestion	41 (77.4%)	32 (57.1%)	0.027 [b]
1 month of age	34 (64.2%)	31 (55.4%)	0.435 [b]
3 months of age	23 (43.4%)	29 (51.8%)	0.445 [b]
Number of times of vomiting milk (times/day)			
1 week after ingestion	0.08 ± 0.02	0.10 ± 0.04	0.492 [a]
1 month of age	0.11 ± 0.03	0.14± 0.06	0.490 [a]
3 months of age	0.03 ± 0.01	0.11 ± 0.06	0.087 [a]
Number of infants with vomiting milk (%)			
1 week after ingestion	16 (30.2%)	14 (25.0%)	0.669 [b]
1 month of age	19 (35.8%)	16 (28.6%)	0.539 [b]
3 months of age	5 (9.4%)	12 (21.4%)	0.114 [b]

Values are represented as the mean ± SEM, $p<0.05$ are statistically significant, [a] Wilcoxon rank sum test, [b] Fisher's exact test

Table S6. Growth of the infant's height, weight, and head circumference at 1 and 3 months after the birth.

		Placebo	M-63	*p*-Value[1]
Height (cm)				
	1 month of age	52.98 ± 0.25	53.44 ± 0.25	0.193
	3 months of age	59.75 ± 0.27	59.99 ± 0.28	0.541
Weight (g)				
	1 month of age	4210.94 ± 76.43	4279.82 ± 66.72	0.497
	3 months of age	6100.39 ± 94.18	6129.84 ± 99.16	0.830
Head circumference (cm)				
	1 month of age	36.55 ± 0.13	36.78 ± 0.16	0.284
	3 months of age	39.65 ± 0.15	39.58 ± 0.25	0.797

All values are represented as the mean ± SEM, [1] Student's t-test

Table S7. Summary of adverse events during the study period.

	Placebo (n = 54)			M-63 (n = 56)			*p*-Value [1]
	Events, n	Infants, n (%)		Events, n	Infants, n (%)		
All disorders n (%)	91	28	(51.9%)	59	24	(42.9%)	0.445
Respiratory disorders	47	14	(25.9%)	33	16	(28.6%)	0.832
Productive cough	9	6	(11.1%)	8	4	(7.1%)	
Nasal congestion	38	14	(25.9%)	25	16	(28.6%)	
Gastrointestinal disorders	30	12	(22.2%)	14	8	(14.3%)	0.329
Diarrhea	7	4	(7.4%)	3	1	(1.8%)	
Oral pain	1	1	(1.9%)	0	0	(0%)	
Gastric hemorrhage	1	1	(1.9%)	0	0	(0%)	
Bloating	0	0	(0%)	1	1	(1.8%)	
Bloody stool	1	1	(1.9%)	0	0	(0%)	
Constipation	3	2	(3.8%)	3	3	(5.4%)	
Vomiting	17	6	(11.1%)	7	5	(8.9%)	
Skin disorders	11	10	(18.5%)	8	7	(12.5%)	0.437
Heat rash	0	0	(0%)	1	1	(1.8%)	
Inflamed skin	1	1	(1.9%)	0	0	(0%)	
Infant eczema	0	0	(0%)	1	1	(1.8%)	
Redness of the skin	1	1	(1.9%)	0	0	(0%)	
Anathema	9	8	(14.8%)	5	4	(7.1%)	
Dry skin	0	0	(0%)	1	1	(1.8%)	
General disorders	2	2	(3.7%)	1	1	(1.8%)	0.615
Fever (over 38 °C)	2	2	(3.7%)	1	1	(1.8%)	
Eye disorders	1	1	(1.9%)	1	1	(1.8%)	1.000
Eye mucus	1	1	(1.9%)	1	1	(1.8%)	
Hepatobiliary disorders	0	0	(0%)	1	1	(1.8%)	1.000
Jaundice	0	0	(0%)	1	1	(1.8%)	
Investigations	0	0	(0%)	1	1	(1.8%)	1.000
T-b, U-b increased	0	0	(0%)	1	1	(1.8%)	

Percentages were calculated as: (number of infants for which symptoms appeared)/(total number of infants during intervention period)*100, [1] Fisher's exact test
